# A comparative study on hypofractionated whole-breast irradiation with sequential or simultaneous integrated boost on different positions after breast-conserving surgery

**DOI:** 10.1038/s41598-021-97520-z

**Published:** 2021-09-09

**Authors:** Ting Yu, Yankang Li, Tao Sun, Min Xu, Wei Wang, Qian Shao, Yingjie Zhang, Jianbin Li, Jinming Yu

**Affiliations:** 1 Key Laboratory of Cancer Prevention and Therapy, Tianjin’s Clinical Research Center for Cancer, Tianjin Medical University, Tianjin Medical University Cancer Institute and Hospital, National Clinical Research Center for Cancer, Tianjin, China; 2 Department of Radiation Oncology, Shandong Cancer Hospital and Institute (Shandong Cancer Hospital), Shandong First Medical University and Shandong Academy of Medical Sciences, 440 Jiyan Road, Jinan, 250117 China; 3 Department of Radiation Physics, Shandong Cancer Hospital and Institute (Shandong Cancer Hospital), Shandong First Medical University and Shandong Academy of Medical Sciences, Jinan, China

**Keywords:** Breast cancer, Cancer, Medical research, Oncology

## Abstract

This study explored the dosimetric difference between hypofractionated whole-breast irradiation (HFWBI) with sequential boost (SEB) and simultaneous integrated boost (SIB) based on supine and prone positions to identify the superior boost mode and superior position. Thirty breast cancer patients eligible for HFWBI after breast-conserving surgery were enrolled. All patients underwent 3DCT simulation scanning in both supine and prone positions. For the SEB-HFWBI plan, the dose prescribed for the planning target volume (PTV) of whole breast (WB) was 2.67 Gy per fraction with a total of 15 fractions, followed by a sequential boost of 3.2 Gy per fraction to the PTV of tumor bed (TB) in 3 fractions. For the SIB-HFWBI plan, the dose prescribed for the PTV of WB was 2.67 Gy per fraction with a total of 15 fractions, with a simultaneously integrated boost of 3.2 Gy per fraction to the PTV of TB with a total of 15 fractions. Regardless of the position, for the PTV of TB, the conformal index (CI) in the SIB-HFWBI plans was greater than those in the SEB-HFWBI plans (*T* = − 8.114, − 8.114; both *P* < 0.05). The CI for the PTV of WB increased significantly in the prone position relative to the supine position in both two plans(*Z* = − 3.340, − 3.501; all *P* < 0.05). The study suggested that prone SIB-HFWBI might be more suitable for postoperative radiotherapy after breast-conserving surgery for early-stage breast cancer patients.

Currently, breast-conserving surgery (BCS) followed by whole-breast irradiation (WBI) is widely accepted as the standard of care for early breast cancer^1–3^. Although conventional fractionation WBI (CFWBI) has remained the main treatment model in China, hypofractionated WBI (HFWBI) has arisen after proposing that the α/β ratio of breast cancer might be as low as approximately 4 in 1989^4^. Two randomized studies found that HFWBI after BCS showed equivalent therapeutic effects and lower acute radiation-induced reactions than CFWBI^5,6^. Moreover, HFWBI provides a shortened time of treatment and improves patient convenience^7^. Hence, HFWBI as a valid alternative for early-stage breast cancer patients after BCS is rapidly replacing CFWBI worldwide.

Both CFWBI and HFWBI are involved in tumor bed (TB) boost since an additional boost after WBI is indispensable for the vast majority of patients after BCS^8^. Furthermore, a boost dose to the TB can improve local control, particularly in young patients with negative prognostic factors for local relapse^9,10^. Frequently, a sequential boost (SEB) to the TB was used, but when the simultaneously integrated boost (SIB) to the TB was used, there was more convenience and superior tolerance for the patient due to shorter treatment time and better dosimetric distribution^11,12^. However, there is no uniform standard for the way (SEB or SIB) and radiation dose fractionation of the tumor bed boost for hypofractionated radiotherapy^13–15^.

Comparative studies on WBI have demonstrated a better dose conformance to the treatment target and a lower dose to the lung in the prone position than in the supine position^16–18^. However, the advantages of HFWBI in different TB boost methods in the supine or prone position have not been established. Therefore, in this study, we performed dosimetric comparisons of the targets and organs at risk (OARs) for HFWBI with the same method of TB boost in the two positions or with different methods of TB boost in the same position, to seek the superior plan and position.

## Methods

### Patient selection

Breast cancer patients eligible for HFWBI^19^ following BCS were enrolled for this study. The oncoplastic BCS was one of the exclusion criterions because of the risk of inconsistent boost delineation and all enrolled patients had ≥ 5 surgical clips fixed to the central bottom and lateral edges of the surgical cavity to mark the TB boundaries. Regional lymph node irradiation was not required for all the enrolled patients. All patients underwent 3DCT simulation scanning both in the supine and prone positions with free breathing on the same day. Moreover, no seroma was observed in the operative cavity during simulation scanning. Full informed consent was obtained for all patients and/or their legal guardians, and the study was approved by the institutional research ethics board of the Shandong Cancer Hospital Ethics Committee and was performed in accordance with relevant guidelines/regulations.

### CT simulation

Patients were scanned for three-dimensional computed tomography (3DCT) simulation under supine and prone positions on a 16-slice computed tomography (CT) scanner (Philips Brilliance Bores CT, Netherlands) with free breathing. For the supine position, the patients were immobilized on a breast board using arm support (with both arms abducted and raised overhead) and knee support. The clinically palpable ipsilateral breast was demarcated with metal wires. The CT simulation in the prone position on a specifically dedicated treatment board was performed in all patients with both arms above their head. The board contained an open aperture on one side to allow for the ipsilateral breast to hang freely away from the chest wall. The CT images were acquired in 3 mm slices from the cricothyroid membrane to 5 cm below the diaphragm. The CT dataset was exported to the Eclipse treatment planning system (Eclipse 15.5, Varian Medical Systems, Palo Alto, CA, USA) for target and OAR delineation and to formulate treatment plans.

### Target definition

The delineation of the target volume and OARs was performed by the same radiation oncologist with over 5 years of experience in breast radiotherapy. On both supine and prone scanning images, the TB was delineated based only on the surgical clips and defined as TB_Supine_ and TB_Prone_, respectively. The clinical target volume (CTV) and planning target volume (PTV) for the TB were created by 5-mm and 10-mm expansion of the TB, respectively, and defined as CTV_Supine-TB_, CTV_Prone-TB_, PTV_Supine-TB_, and PTV_Prone-TB_. The CTV for the whole breast (WB) included the glandular breast tissue of the ipsilateral breast and was defined as CTV_Supine-WB_ and CTV_Prone-WB_. The PTV for the WB was the CTV for the WB plus a 5-mm margin and defined as PTV_Supine-WB_ and PTV_Prone-WB_, respectively. Moreover, the CTV for the WB was limited to the glandular-pectoral muscle wall interface and 5 mm from the skin surface, including the CTV for the TB. The target volume, as well as all organs at risk, such as heart, lung and contralateral breast were contoured according to the Radiation Therapy Oncology Group (RTOG) delineation guidelines for adjuvant radiotherapy of early breast cancer^20^. All delineation of the targets was determined using the same clinical criteria, whether in the supine or prone position.

### Treatment planning

**Figure 1:**
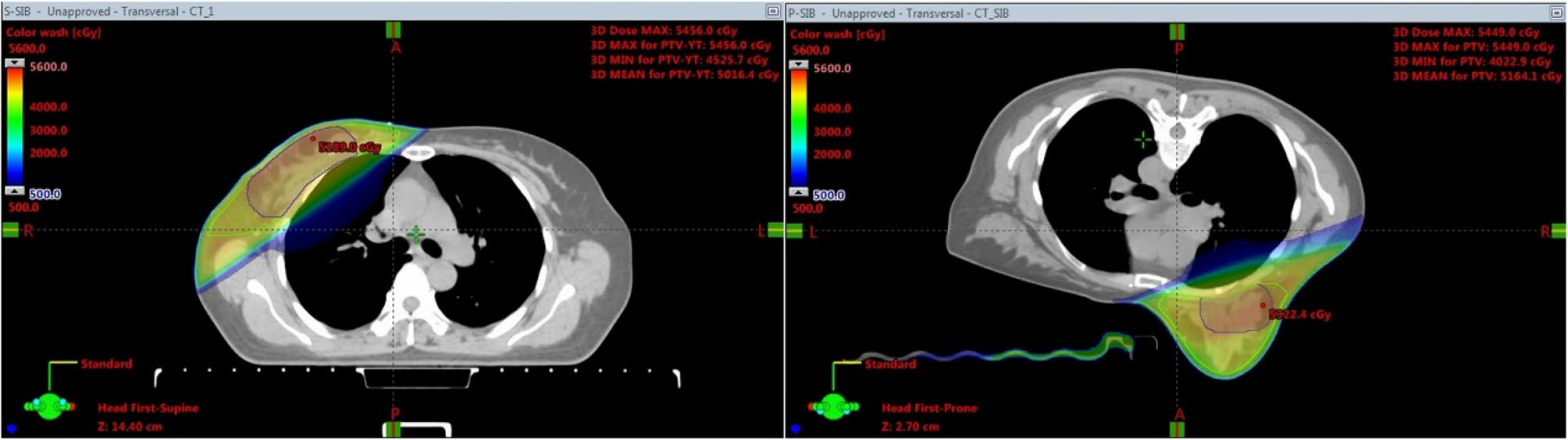
The picture of target volumes and isodose distribution based on supine and prone position for SIB-HFWBI.

**Figure 2:**
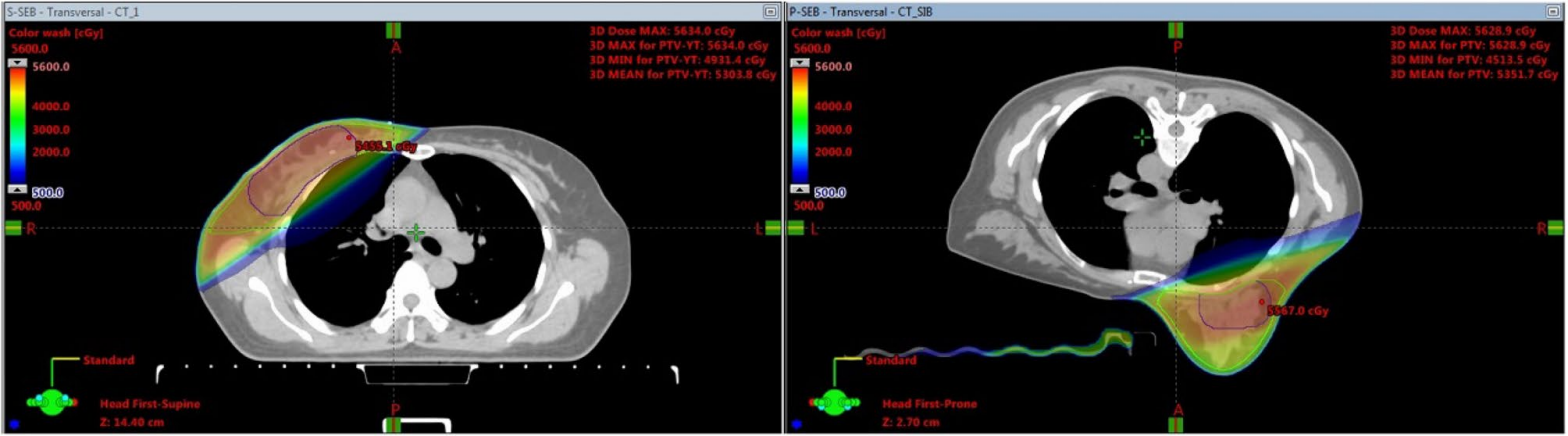
The picture of target volumes and isodose distribution based on supine and prone position for SEB-HFWBI.

For each patient, four different plans, SIB-HFWBI and SEB-HFWBI in both supine and prone positions, were generated. All treatment plans were performed in VARIAN’s ECLIPSE TPS Version 15.5 (Anisotropic Analytical Algorithm calculation model) using field in field technique with a 6-MV photon beam. For the SIB-HFWBI plan, the dose prescribed for the PTV of the WB was 2.67 Gy per fraction with a total of 15 fractions, with a simultaneously integrated boost of 3.2 Gy per fraction to the PTV of the TB with a total of 15 fractions (Fig. 1). For the SEB-HFWBI plan, the dose prescribed for the PTV of the WB was 2.67 Gy per fraction with a total of 15 fractions, followed by a sequential boost of 3.2 Gy per fraction to the PTV of the TB in 3 fractions (Fig. 2). In addition, the criteria of the plans were to ensure that at least 95% of the PTV received the prescription dose. Optimization was addressed to reduce both the dose for the IPSL and the heart. In the supine treatment planning, patients were treated with two opposing tangential fields for the prescribed dose to be delivered to the WB. To reduce the IPSL volume as much as possible, 4–6 segmented fields were set up to adjust the homogeneity of the target volume. The field angle of the TB was the same in both the SEB-HFWBI and SIB-HFWBI plans. In the prone treatment planning, two opposing tangential fields were also set up for the prescribed dose to be delivered to the WB. The field angle was chosen to avoid exposure to the contralateral breast as the primary consideration and to minimize the ipsilateral irradiated lung volume. Moreover, 4–6 segmented fields were also added to adjust the homogeneity of the target volume in the prone setup. In the SIB-HFWBI plan, the entire breast and TB were simultaneously irradiated, while in the SEB setup, the treatment plan for total breast and TB were superimposed and calculated.

### Dosimetric evaluation

Dose-volume histogram (DVH) parameters for the PTV, heart, IPSL, and contralateral breast were calculated for each plan in all patients. The conformal index (CI) and homogeneity index (HI) were evaluated for the PTV. The V100% means the PTV coverage percent of the 100% prescribed dose line in the treatment plan.

CI was defined as follows:

$${\text{CI}} = \frac{{{\text{Ref}}.\;{\text{isodose}}\;{\text{volume}}\;{\text{of}}\;{\text{the}}\;{\text{PTV}}}}{{{\text{PTV}}}} \times \frac{{{\text{Ref}}.\;{\text{isodose}}\;{\text{volume}}\;{\text{of}}\;{\text{the}}\;{\text{PTV}}}}{{{\text{Ref}}.\;{\text{isodose}}\;{\text{volume}}.{ }}}$$

where Ref. isodose volume of the PTV represents the absolute volume of the PTV that is covered by the prescribed dose and Ref. isodose volume represents the absolute volume covered by the prescribed dose^21^.

HI was defined as follows:

$${\text{HI}} = \frac{{{\text{D}}2 - {\text{D}}98}}{{{\text{D}}50}}$$

where D_2_ and D_98_ represent the doses covering 2% and 98% of the PTV, respectively^22^.

The IPSL and heart were evaluated using the mean dose (D_mean_) and the volumes that received ≥ 5 Gy, 10 Gy, 20 Gy, 30 Gy and 40 Gy (V_5_, V_10_, V_20,_ V_30_ and V_40_, respectively). The contralateral breast was evaluated using the D_mean_ and D_2_. The MU represented the monitor units in the treatment plan.

### Statistical methods

Statistical analysis was performed with SPSS 19.0 software (IBM Corporation, Armonk, NY, USA). The data that did not follow a normal distribution were analyzed by the Wilcoxon signed-rank test and are described using medians and ranges. Data that followed a normal distribution were analyzed by paired-samples t-tests and are described using means and standard deviations. The Wilcoxon signed-rank test was used to compare the dosimetric parameters of the targets and IPSL for HFWBI with different methods of TB boost in the same position. Our study performed dosimetric comparisons of the heart between the SEB-HFWBI and SIB-HFWBI regimens in the same position via paired-samples t-tests. Data were considered statistically significant at *P* < 0.05.

## Results

### Patient characteristics

Our study analyzed thirty patients treated with HFWBI following BCS for early-stage breast cancer between July 2018 and December 2019. The median age was 46 (ranging from 30 to 60). Patients had stage I or II (TIN0M0-T2N0M0) breast cancer according to the 2009 7th edition of the American Joint Committee on Cancer. Fourteen of the 30 patients had left-sided breast cancer, and the remaining sixteen had right-sided breast cancer. Patients underwent lumpectomy with sentinel lymph node dissection (SLND) or axillary lymph node dissection (ALND) and had ensured tumor-negative margins during a single operation. The characteristics of the study population are displayed in Table 1.

**Table 1:** Patient and tumor characteristics.

Variable	Value
**Age, years**	
Median	46
Range	30–60
**Tumor size**	
≥ 10 mm < 20 mm	16
≥ 20 mm	14
**Breast side**	
Left	14
Right	16
**Breast volume**	
< 750mm^3^	23
≥ 750mm^3^	7
**Localization of the TB**	
UOQ	14
LOQ	5
Central portion of the breast	4
UIQ	4
LIQ	3
**Tumor characteristics**	
Ductal carcinoma in situ	2
Invasive ductal carcinoma	23
Invasive lobular carcinoma	2
Cribriform carcinoma	1
Mucinous carcinoma	2

*UOQ* upper outer quadrant, *LOQ* lower outer quadrant, *UIQ* upper inner quadrant, *LIQ* lower inner quadrant.

### Dosimetric comparisons of the targets between the SEB-HFWBI and SIB-HFWBI plans on the same position

Table 2 shows that the dosimetric parameters, including D_2_, D_98_ and V_100%_for PTV_TB_ and V_100%_ for PTV_WB_ were all significantly higher for the SEB-HFWBI plan than for the SIB-HFWBI plan based on the same position (all *P* < 0.05). The CIs for PTV_TB_ and PTV_WB_ were significantly lower in the SEB-HFWBI plan than in the SIB-HFWBI plan in both the supine and prone positions (supine: *T* = − 8.114, − 13.356; prone: *T* = − 8.114, − 13.356; all *P* < 0.05, see Table 3 for details). Furthermore, regardless of patient position, the HI of PTV_TB_ was significantly better with the SIB approach than with the SEB approach (supine: *Z* = − 6.552, *P* = 0.000; prone: *Z* = − 6.552; *P* = 0.000).

**Table 2:** Dosimetric evaluation of the targets for the SEB-HFWBI and SIB-HFWBI plans inthe same position (median).

Parameters	SEB-HFWBI	SIB-HFWBI	*Z*-value	*P*-value
**Supine**				
*PTV*_*Supine*_*-*_*TB*_				
D2 (Gy)	54.85 (53.71–56.46)	53.20 (52.22–54.91)	− 4.782	0.000
D98 (Gy)	50.89 (44.22–52.96)	48.77 (42.59–51.26)	− 4.782	0.000
Dmean (Gy)	53.11 (51.78–54.79)	51.04 (49.77–52.66)	− 4.762	0.000
V100%	99.35 (95.80–99.97)	98.55 (95.50–99.80)	− 4.280	0.000
*PTV*_*Supine-WB*_				
V100%	98.10 (96.40–99.60)	96.55 (86.30–98.50)	− 4.621	0.000
*PTV*_*Supine-WB*_*-PTV*_*Supine-TB*_				
D2 (Gy)	54.80 (53.80–56.09)	52.68 (50.56–54.17)	− 4.541	0.000
D98 (Gy)	33.08 (28.81–36.34)	33.35 (28.75–36.20)	− 0.625	0.532
V100%	12.65 (4.00–25.70)	8.60 (2.60–14.70)	− 4.762	0.000
**Prone**				
*PTV*_*Prone-TB*_				
D2 (Gy)	55.05 (53.71–56.75)	53.57 (52.18–55.28)	− 4.349	0.000
D98 (Gy)	51.53 (43.06–53.39)	49.18 (43.44–51.76)	− 3.315	0.000
Dmean (Gy)	53.57 (51.42–54.24)	51.95 (50.13–57.98)	− 3.868	0.000
V100%	99.30 (92.00–99.90)	98.40 (93.70–99.90)	− 2.987	0.003
*PTV*_*Prone-WB*_				
V100 (%)	98.30 (86.50–100.00)	96.55 (95.10–98.40)	− 3.961	0.000
*PTV*_*Prone-WB*_*-PTV*_*Prone-TB*_				
D2 (Gy)	54.84 (54.26–55.70)	52.89 (51.62–54.02)	− 4.541	0.000
D98 (Gy)	33.07 (17.49–38.33)	32.61(15.90–52.92)	− 0.144	0.885
V100%	20.40 (5.20–32.40)	13.55 (3.80–36.20)	− 4.196	0.000

*TB* tumor bed, *PTV*_*TB*_ planning target volume for the TB, *WB* whole breast, *PTV*_*WB*_ planning target volume for the WB, *PTV*_*WB*_*-PTV*_*TB*_ the target volume obtained by subtracting the PTV_TB_ from the PTV_WB_, *SIB* simultaneous integrated boost, *SEB* sequential integrated boost, *HFWBI* hypofractionated whole-breast irradiation, *D2* doses covering 2% of the PTV, *D98* doses covering 98% of the PTV, *Dmean* mean dose, *V100%* the PTV coverage percent of the 100% prescribed dose line including PTV_TB_, PTV_WB_, PTV_WB_-PTV_TB_ in the treatment plan under supine and prone positions.

**Table 3:** Comparison of the CI and HI for the targets between SEB-HFWBI and SIB-HFWBI plans in the same position (mean).

Parameters	SEB-HFWBI	SIB-HFWBI	*T*-value	*P*-value
**Supine**				
*PTV*_*Supine-TB*_				
CI	0.48 ± 0.06	0.58 ± 0.07	− 8.114	0.000
HI	0.09 ± 0.01	0.10 ± 0.01	− 6.552	0.000
*PTV*_*Supine-WB*_				
CI	0.63 ± 0.07	0.66 ± 0.06	− 13.356	0.000
*PTV*_*Supine-WB*_*-PTV*_*Supine-TB*_				
HI	0.31 ± 0.02	0.19 ± 0.02	34.467	0.000
**Prone**				
*PTV*_*Prone-TB*_				
CI	0.46 ± 0.08	0.54 ± 0.09	− 8.114	0.000
HI	0.09 ± 0.02	0.10 ± 0.01	− 6.552	0.000
*PTV*_*Prone-WB*_				
CI	0.68 ± 0.07	0.71 ± 0.07	− 13.356	0.000
*PTV*_*Prone-WB*_*-PTV*_*Prone-TB*_				
HI	0.31 ± 0.02	0.21 ± 0.02	34.467	0.000

*TB* tumor bed, *PTV*_*TB*_ planning target volume for the TB, *WB* whole breast, *PTV*_*WB*_ planning target volume for the WB, *SIB* simultaneous integrated boost, *SEB* sequential integrated boost, *HFWBI* hypofractionated whole-breast irradiation, *CI* conformal index, *HI* homogeneity index, *PTVsupine-*_*WB*_*-PTVsupine-*_*TB*_ the target volume obtained by subtracting the PTV_TB_ from the PTV_WB_ in supine position, *PTVprone-*_*WB*_*-PTVprone-*_*TB*_ the target volume obtained by subtracting the PTV_TB_ from the PTV_WB_ in prone position.

### Comparison of dosimetric parameters of the OARs between the SEB-HFWBI and SIB-HFWBI plans in the same position

The IPSL dose parameters (D_mean_, V_5_, V_10_, V_20_, V_30_, V_40_) showed significantly lower averages for the SIB-HFWBI plan than for the SEB-HFWBI plan in both the supine and prone positions (all *P* < 0.05, see Table 4 for details). The values for heart dose parameters, including D_mean_, V_5_, V_10_, V_20_, V_30_, and V_40,_ in left-sided breast cancer patients treated with the SEB-HFWBI plan were significantly higher than in those treated with the SIB-HFWBI plan in the same position (all *P* < 0.05, see Table 4 for details). In both the supine and prone position, the D_mean_ to the heart showed no statistically significant differences between the SIB-HFWBI and SEB-HFWBI plans in the right-sided breast cancer patients (*Z* = − 1.518, − 1.741, *P* = 0.067, 0.076). In the SIB-HFWBI regimen, D_2_ and D_mean_ to the contralateral breast was significantly lower than that in the SEB-HFWBI regimen in both the supine and prone positions (S: *Z* = − 3.252, − 3.658; *P* = 0.001, 0.000; *P*: *Z* = − 3.252, − 3.658; *P* = 0.001, 0.000). The SIB setup indeed revealed fewer MUs than the SEB setup in both the supine and prone positions, and the differences were statistically significant (*Z* = − 4.783, 4.783; *P* = 0.000, 0.000, see Table 5 for details).

**Table 4:** Dosimetric evaluation of the IPSL and heart to left breast cancer patients between the SEB-HFWBI and SIB-HFWBI plans in the same position (mean).

Parameters	SEB-HFWBI	SIB-HFWBI	*T*-value	*P*-value
**Ipsilateral lung supine**				
V_5_ (%)	31.52 ± 5.64	30.91 ± 5.65	4.479	0.000
V_10_ (%)	22.71 ± 5.46	22.45 ± 5.47	6.632	0.000
V_20_ (%)	18.20 ± 5.21	18.06 ± 5.22	5.771	0.000
V_30_ (%)	15.11 ± 4.99	14.89 ± 5.02	7.550	0.000
V_40_ (%)	6.16 ± 3.83	4.41 ± 3.28	9.766	0.000
D_mean_ (Gy)	9.24 ± 1.98	8.96 ± 1.93	9.251	0.000
**Prone**				
V_5_ (%)	13.12 ± 6.95	12.76 ± 6.89	4.479	0.000
V_10_ (%)	8.70 ± 5.88	8.54 ± 5.83	6.632	0.000
V_20_ (%)	5.10 ± 3.95	4.89 ± 3.86	5.771	0.000
V_30_ (%)	3.15 ± 2.83	3.00 ± 2.76	7.550	0.000
V_40_ (%)	1.28 ± 1.55	0.79 ± 1.23	9.766	0.000
D_mean_ (Gy)	3.57 ± 1.81	3.42 ± 1.74	9.251	0.000
**Heart in left-sided patients supine**				
D_mean_ (Gy)	5.38 ± 2.07	5.29 ± 2.06	4.752	0.000
V_5_ (%)	16.18 ± 6.46	15.88 ± 6.38	4.364	0.001
V_10_ (%)	11.98 ± 5.60	11.90 ± 5.60	3.294	0.006
V_20_ (%)	9.70 ± 5.06	9.65 ± 5.09	2.270	0.041
V_30_ (%)	7.94 ± 4.53	7.89 ± 4.54	2.876	0.013
V_40_ (%)	2.88 ± 1.91	2.33 ± 1.75	4.125	0.001
**Prone**				
D_mean_ (Gy)	5.20 ± 2.30	5.04 ± 2.26	4.734	0.000
V_5_ (%)	18.10 ± 9.00	17.71 ± 8.91	5.498	0.000
V_10_ (%)	13.34 ± 8.21	13.18 ± 8.16	4.112	0.000
V_20_ (%)	9.10 ± 5.67	8.88 ± 5.57	3.231	0.003
V_30_ (%)	5.06 ± 3.69	4.91 ± 3.62	4.380	0.001
V_40_ (%)	2.22 ± 2.09	1.73 ± 1.75	2.902	0.012

*SIB* simultaneous integrated boost, *SEB* sequential integrated boost, *HFWBI* hypofractionated whole-breast irradiation, *V*_*5*_ the volumes that received ≥ 5 Gy, *V*_*10*_ the volumes that received ≥ 10 Gy, *V*_*20*_ the volumes that received ≥ 20 Gy, *V*_*30*_ the volumes that received ≥ 30 Gy, *V*_*40*_ the volumes that received ≥ 40 Gy, *D*_*mean*_ the mean dose.

**Table 5:** Dosimetric evaluation for the contralateral breast and MU between the SEB-HFWBI and SIB-HFWBI treatment plans in the same position (median).

Parameters	SEB-HFWBI	SIB-HFWBI	*Z*-value	*P*-value
**Supine**				
*Contralateral breast*				
D_2_ (Gy)	1.37 (0.00–16.54)	1.36 (0.00–16.56)	− 3.252	0.001
Dmean (Gy)	0.22 (0.00–1.07)	0.21 (0.00–1.07)	− 3.658	0.000
MU	6321 (6045–6861)	5730 (5430–6255)	− 4.782	0.000
**Prone**				
*Contralateral breast*				
D_2_ (Gy)	1.38 (0.00–13.60)	1.23 (0.00–13.11)	− 3.252	0.001
Dmean (Gy)	0.33 (0.00–2.87)	0.30 (0.00–2.78)	− 3.658	0.000
MU	6346 (5895–8586)	5797 (5340–7955)	− 4.783	0.000

*SIB* simultaneous integrated boost, *SEB* sequential integrated boost, *HFWBI* hypofractionated whole-breast irradiation, *D*_*2*_ doses covering 2% of the PTV, *D*_*98*_ doses covering 98% of the PTV, *Dmean* mean dose, *MU* monitor units for whole treatment.

### Dosimetric comparison of the targets and OARs for HFWBI with the same TB boost in two different positions

For both the SIB-HFWBI and SEB-HFWBI plans, the CI for the PTV_WB_ increased slightly in the prone position relative to the supine position (*Z* = − 3.340, − 3.501; all *P* < 0.05, see Table 6 for details). Moreover, for the IPSL, the *D*_mean_, *V*_5_, *V*_10_, and *V*_20_ obtained in the prone position were all significantly lower than those obtained in the supine position in both the SIB-HFWBI plan and SEB-HFWBI plan (SIB: *Z* = − 4.782, − 4.704, − 4.782, − 4.783; SEB: Z = − 4.782, − 4.782, − 4.782, − 4.782; all *P* = 0.000). Regardless of the SEB or SIB approach, no significant differences in the *D*_mean_ to the heart were evident between the supine and prone positions in the left-sided breast cancer patients (T = 0.278, 0.393; *P* = 0.786, 0.701).

**Table 6:** Dosimetric evaluation of the targets for the SEB-HFWBI or SIB-HFWBI treatment plan in the supine and prone positions (median).

Parameters	Supine	Prone	*Z*-value	*P*-value
**SEB-HFWBI**				
*PTV*_*TB*_				
Dmean (Gy)	53.11 (51.78–54.79)	53.57 (51.42–54.24)	− 2.478	0.013
V_100%_	99.35 (95.80–99.97)	99.30 (92.00–99.90)	− 0.577	0.564
*PTV*_*WB*_				
V_100%_	98.10 (96.40–99.60)	98.30 (86.50–100.00)	− 0.748	0.455
CI	0.63 (0.47–0.75)	0.69 (0.50–0.82)	− 3.340	0.001
*PTV*_*WB*_*-PTV*_*TB*_				
V_100%_	12.65 (4.00–25.70)	20.4 (5.20–32.40)	− 3.908	0.000
**SIB-HFWBI**				
*PTV*_*TB*_				
Dmean (Gy)	51.04(49.77–52.66)	51.95 (50.13–57.98)	− 2.036	0.021
V_100%_	98.55 (95.50–99.80)	98.40 (93.70–99.90)	− 0.068	0.946
*PTV*_*WB*_				
V_100%_	96.55 (86.30–98.50)	96.55 (95.10–98.40)	− 0.216	0.829
CI	0.66 (0.50–0.77)	0.72 (0.52–0.83)	− 3.501	0.000
*PTV*_*WB*_*-PTV*_*TB*_				
V_100%_	8.60 (2.60–14.70)	13.55 (3.80–36.20)	− 4.165	0.000

*TB* tumor bed, *PTV*_*TB*_ planning target volume for TB, *WB* whole breast, *PTV*_*WB*_ planning target volume for WB, *PTV*_*WB*_*-PTV*_*TB*_ the target volume obtained by subtracting the PTV_TB_ from the PTV_WB_, *SIB* simultaneous integrated boost, *SEB* sequential integrated boost, *HFWBI* hypofractionated whole-breast irradiation, *CI* conformal index, *Dmean* mean dose, *V100%* the PTV coverage percent of the 100% prescribed dose line including PTV_TB,_ PTV_WB,_ PTV_WB_-PTV_TB_ in the treatment plan under supine and prone positions.

## Discussion

A commonly used regimen involves WBI after BCS to a dose of 45–50 Gy over 5 weeks, with a sequential boost delivered to the TB, which again prolongs the overall treatment time by 1–2 weeks. Although the normal fractionation scheme of WBI is widely accepted, approximately 15–20% of BCS patients eventually choose to give up on radiotherapy due to a lengthy treatment course^23,24^. As the results from multiple randomized studies have been gradually published^25–27^, HFWBI in 15 or 16 fractions is slowly replacing normal fractionation schemes for WBI worldwide. Indeed, HFWBI as an alternative to the CFWBI regimen has become a superior choice for early breast cancer patients after BCS, which substantially increases patient convenience because of shortened treatment duration and a reduction in cost. Furthermore, randomized controlled trials comparing HFWBI with CFWBI showed a slight reduction in acute toxicity and significantly better cosmetic outcomes^19,28^.

A TB boost should also be an essential part of the standard setup for HFWBI. However, the relevant studies^25–27^ that laid the foundation for the safety and equivalence of HFWBI had no uniform agreement regarding the TB boost after HFWBI. The optimal dose, fractionation schedule, delivery method, and timing of the boost remain undefined. A Canadian multicenter, prospective, randomized trial of early-stage breast cancer patients reported the equivalence of HFWBI (a dose of 42.5 Gy in 16 fractions) to CFWBI (a dose of 50 Gy in 25 fractions)^27^. However, a boost to the TB was not included in this trial. The UK START Trial A delivered 10 Gy to the TB in five daily fractions after HFWBI sequentially^25,26^. Recent studies have explored the regimen of TB boost in HFWBI^14,29^. Gupta et al.^14^ reported the results of a phase 2 HFWBI randomized trial with a follow-up of 5 years. They delivered a WB dose of 36.63 Gy in 11 fractions of 3.33 Gy, followed by a TB boost of 13.32 Gy in 4 fractions of 3.33 Gy. The results of the 5-year follow-up showed that the locoregional control reached 97.7%, the rate of excellent breast cosmesis reached 95% and the acute and late toxicity rates were relatively low. A randomized controlled trial comparing CF-WBI with HFWBI verified that the overall rates of any physician-assessed acute toxic effects of grade 2 or higher or grade 3 or higher were lower with HFWBI than with CFWBI (47% and 78%, *P* < 0.001)^29^. Schmeel et al.^30^ reported similar results in their randomized controlled trial. In terms of the comparison between sequential and simultaneous integrated boost, the results of a phase III randomized study conducted by Paelinck et al.^15^ demonstrated that grade 2/3 dermatitis was significantly more frequent in the SEB-HFWBI arm and that the incidence of edema was lower in the SIB-HFWBI arm. But the latest data showed the physician-assessed two-years toxicity and photographic analysis were not significantly different between SIB and SEB treatment arms^31^. Nevertheless, Onal et al.^32^ demonstrated that the SIB technique showed better target-volume dose distribution and better sparing heart in volumetric-modulated arc therapy and helical-tomotherapy compared to the SEB technique. Consequently, even though the above studies have shown that HFWBI is superior to CFWBI, the controversy between the SIB and SEB still remained. Afterwards, in our study, when comparing the values for OAR dose parameters including the IPSL, the contralateral breast and heart in left-sided breast cancer patients, significantly lower averages were found for the SIB-HFWBI plan.

To the best of our knowledge, our study on the comparison of dosimetric parameters between SEB-HFWBI and SIB-HFWBI in prone and supine positions is the first to address this topic using FIMRT. In this study, we first compared the differences between SEB and SIB plans in the same position. The results showed that the CI of PTV_TB_ and PTV_WB_ in SIB plans were better than that in the SEB plans, and the PTV_TB_ in the SIB plan had better dose homogeneity compared to the SEB plan. For organs at risk, the SIB plans significantly reduced the dose to the ipsilateral lung, heart and contralateral breast. The SIB plans significantly reduced MUs, which could reduce machine wastage. In conclusion, the SIB plans showed better dosimetric advantages over the SEB plans in both supine and prone position. Van Parijs et al.^33^ compared SEB and SIB plans of 10 patients with breast cancer in supine position, the results confirmed the dosimetric advantages of SIB for breast irradiation, even when compared to an advanced and highly conformal sequential technique. The result was the same as our study.

Then, we compared the treatment plans for different positions. The results showed that, for both the SIB-HFWBI and SEB-HFWBI plans, the CI for the PTV_WB_ was superior in the prone position than in the supine position. Furthermore, for the IPSL, the dose parameters obtained in the prone position were all significantly lower than those obtained in the supine position. However, for the left-sided breast cancer patients, we verified that the variance in the heart dose parameters between supine and prone positions was not statistically significant with either the SEB-HFWBI or SIB-HFWBI approach. This may be because in the prone position, the heart droops and enters the irradiation field. In fact, several studies have clarified the dosimetric advantages of CFWBI in prone position^13,34–36^. Bergom et al.^36^ and Alonso-Basanta et al.^37^ reported that the dose homogeneity in prone WBI was improved and that the high dose distribution to the target was also reduced accordingly. Osa et al.^38^ also indicated that the advantages of prone SIB-HFWBI were the significantly reduced in-field volume of the IPSL and heart. Controversy exists regarding supine and prone positions in terms of the irradiated dose to the heart. Lymberis et al.^39^ indicated that prone positioning reduced the in-field heart volume in the majority (87%) of left-sided breast cancer patients. However, the results of previous study^38^ concluded that no significant difference in the in-field volume of the heart was observed between the supine and prone positions, which was consistent with our findings. Furthermore, Kim et al.^40^ suggested that the breast target volume for patients with small breasts (< 750 cm^3^) were no difference between the supine and prone positions. However, Kirby et al.^35^ also found that about two-thirds of breast cancer patients could benefit from the prone irradiation, especially for the protection to the heart and the left coronary artery. And further analysis showed that only a whole breast CTV > 1000 cm^3^ was associated with improved cardiac dosimetry under the prone position^35^. In our study, the enrolled patients with breast volumes less than 750 cm^3^ comprised 76% of all patients. Meanwhile, only 10% of the women had breast volumes > 1000 cm^3^. Therefore, even though the heart droops in the prone position, the irradiation field entered in heart was similar to the supine position.

In our study, deep inspiration breath hold (DIBH) was not used. To further reduce the dose to the heart and lung, DIBH could be used. Mulliez et al.^41^ verified that the ability and feasibility of prone deep inspiration breath hold to decrease the in-field volume of heart and lung for left-sided WBI. For prone positioning, there is a problem about postural repeatability. Although Deseyne et al.^42^ demonstrated that a newly developed crawl couch could improve precision and comfort and reduce set-up errors compared to the standard prone breast board in prone-WBI. Lakosi et al.^43^ analyzed respiratory motion of surgical clips, chest wall (CW) and the anterior displacement of the heart, results showed that prone position significantly reduced respiration related CW and surgical clip movements but increased anterior heart displacement. The study recommended daily online correction to maximize the heart protection effect in prone position.

## Conclusion

Regardless of the supine or prone position, SIB-HFWBI offered more appropriate target coverage and lower doses to OARs, especially the IPSL, contralateral breast and heart, in left breast cancer patients. For both the SEB-HFWBI plan and SIB-HFWBI plans, the prone treatment showed a better dose conformance to the treatment target and a lower dose to the lung in the prone position than in the supine position. In summary, our study suggested that prone SIB-HFWBI might be more suitable for postoperative radiotherapy after breast-conserving surgery for early-stage breast cancer patients.

## Abbreviations

HF-WBI: Hypofractionated whole-breast irradiation
CFWBI: Conventional fractionation whole-breast irradiation
WBI: Whole breast radiation
3DCT: Three-dimensional computed tomography
BCS: Breast-conserving surgery
FIMRT: Forward intensity modulated radiation therapy
SIB: Simultaneous integrated boost
SEB: Sequential integrated boost
TB: Tumor bed
CTV: Clinical target volume
PTV: Planning target volume
PTV_TB_: Planning target volume for the TB
WB: Whole breast
PTV_WB_: Planning target volume for the WB
OARs: Organs at risk
SLND: Sentinel lymph node dissection
ALND: Axillary lymph node dissection
UOQ: Upper outer quadrant
LOQ: Lower outer quadrant
UIQ: Upper inner quadrant
LIQ: Lower inner quadrant
CI: Conformal index
HI: Homogeneity index
V5: The volumes that received ≥ 5 Gy
V10: The volumes that received ≥ 10 Gy
V20: The volumes that received ≥ 20 Gy
V30: The volumes that received ≥ 30 Gy
V40: The volumes that received ≥ 40 Gy
Dmean: The mean dose
D2: The doses covering 2% of the PTV
D98: The doses covering 98% of the PTV
V100%: The PTV coverage percent of the 100% prescribed line in the treatment plan
MU: Monitor unit
DVH: Dose-volume histogram
CW: Chest wall
DIBH: Deep inspiration breath hold
PTV_WB_-PTV_TB_: The target volume obtained by subtracting the PTV_TB_ from the PTV_WB_
